# Concise Cascade Methods for Transgenic Rice Seed Discrimination using Spectral Phenotyping

**DOI:** 10.34133/plantphenomics.0071

**Published:** 2023-07-28

**Authors:** Jinnuo Zhang, Xuping Feng, Jian Jin, Hui Fang

**Affiliations:** ^1^ Department of Agricultural and Biological Engineering, Purdue University, West Lafayette, IN 47907, USA.; ^2^ College of Biosystems Engineering and Food Science, Zhejiang University, Hangzhou, China.; ^3^ Huzhou Institute of Zhejiang University, Huzhou, China.

## Abstract

Currently, the presence of genetically modified (GM) organisms in agro-food markets is strictly regulated by enacted legislation worldwide. It is essential to ensure the traceability of these transgenic products for food safety, consumer choice, environmental monitoring, market integrity, and scientific research. However, detecting the existence of GM organisms involves a combination of complex, time-consuming, and labor-intensive techniques requiring high-level professional skills. In this paper, a concise and rapid pipeline method to identify transgenic rice seeds was proposed on the basis of spectral imaging technologies and the deep learning approach. The composition of metabolome across 3 rice seed lines containing the *cry1Ab/cry1Ac* gene was compared and studied, substantiating the intrinsic variability induced by these GM traits. Results showed that near-infrared and terahertz spectra from different genotypes could reveal the regularity of GM metabolic variation. The established cascade deep learning model divided GM discrimination into 2 phases including variety classification and GM status identification. It could be found that terahertz absorption spectra contained more valuable features and achieved the highest accuracy of 97.04% for variety classification and 99.71% for GM status identification. Moreover, a modified guided backpropagation algorithm was proposed to select the task-specific characteristic wavelengths for further reducing the redundancy of the original spectra. The experimental validation of the cascade discriminant method in conjunction with spectroscopy confirmed its viability, simplicity, and effectiveness as a valuable tool for the detection of GM rice seeds. This approach also demonstrated its great potential in distilling crucial features for expedited transgenic risk assessment.

## Introduction

Cereals are among the most substantial cultivated plants for the basic global food supply and biofuel manufacturing [1]. To further increase cereals’ production amount and quality, numerous factors that can risk cereals’ normal growth during cultivation should be carefully considered and controlled. Benefiting from genetically modified (GM) technology, the resistance capability of crops, as well as the nutritive value, to adverse environmental circumstances including biotic and abiotic stress is improved [2]. However, GM foods including cereals are surrounded by controversy over biosafety, despite there being deficient evidence of hazards associated with GM crops and extensive safety assessments before their release. Clear labeling and traceability of GM-related products are necessary to alleviate concerns and increase customer trust. Various analytical methodologies that can precisely monitor and verify the presence and amount of GM organisms have been developed, such as the polymerase chain reaction method [3,4], lateral flow strip [5,6], enzyme-linked immunosorbent assays [7], and so on. Nevertheless, these methods involve complicated sample preparation procedures such as extracting DNA or protein, and highly professional experience is also required in the subsequent testing. In this respect, a more concise and rapid way to discriminate against the GM organism without complex and laborious pretreatment might be advantageous with the expansion of the amount of the evaluated sample.

Among the innovative plant phenotyping techniques, spectroscopic methodologies in the range of near infrared (NIR) [8] and terahertz [9] have been deployed to fulfill the need for rapid organism detection. Hacisalihoglu et al. [10] have utilized NIR spectra to evaluate the protein and oil content in the pea seed and achieved a high prediction accuracy for protein. Liu et al. [11] investigated the starch content in corn kernel using NIR hyperspectral images, and the determination coefficient reached 0.96. Although the DNA structure changes cannot be directly recognized by these spectral techniques, it is possible to measure and quantify the dominant structural variations brought by the changes in the DNA structure of GM products [12]. The changes in substance composition caused by exogenous DNA insertion can be manifested through different reactions in the vibration of chemical bonds, especially the vibration of hydrogen-related functional groups (H–N, H–C, O–H, and S–H) in NIR spectra. This provides molecular-level references for identifying GM rice seeds based on their unique spectral signatures. The terahertz spectra correspond to the energy associated with molecular vibrations and low-frequency collective motions, providing valuable information about the molecular structure and dynamics [13]. Moreover, the terahertz spectra exhibit characteristic absorption features that are remarkably sensitive to specific molecular vibrations. By analyzing the absorption features, detailed insights into molecular vibrations and interactions can be obtained at a larger level than NIR spectra. However, direct analysis of NIR or terahertz spectra poses substantial challenges since they involve the fusion of multiple signal characteristics. It is necessary to use feature analysis algorithms with chemometrics techniques to untangle the hidden valuable signals.

Machine learning algorithms excel at multivariate feature analysis, which makes them exceptionally well suited for processing and analyzing spectral information. Earlier studies have demonstrated the successful application of spectroscopy and various machine learning models to discriminant GM organisms [14–16]. Feng et al. [17] applied NIR spectra extracted from hyperspectral images to differentiate GM maize kernel together with partial least-squares discriminant analysis (PLS-DA) with a prediction accuracy of 98.17 %. Their founding provided solid proof for the feasibility of NIR spectra, although only a single genotype was introduced. da Mata et al. [18] collected both NIR spectra of the transgenic cotton seeds and got PLS-DA prediction errors for the prediction set of 2.23% for NIR. Liu et al. [16] tried to discern GM *Bacillus thuringiensis* (Bt) rice seeds using terahertz (0 to 5 THz) and a random forest model with an identification accuracy of 96.67%. Nonetheless, these studies have only investigated a pair of transgenic samples and did not consider the impact of spectral interclass shift caused by multiple genotypes. Moreover, in the theoretical part, the mechanism of the spectrum-based GM classification and the intrinsic metabolic variability induced by these GM traits was seldom explored. In addition, in the methodology part, the high-dimensional nature of spectral data introduces challenges for data analysis, often referred to as the “curse of dimensionality”. To overcome this challenge, it is crucial to use effective feature extraction techniques that can extract meaningful and relevant information from high-dimensional data for rapid and concise discrimination. In those studies, several traditional wavelength selection algorithms such as the principal components (PC) analysis (PCA) [14,16], successive projection algorithm (SPA) [15], and competitive adaptive reweighted sampling [17] were applied to get the characteristic bands. These dimensionality reduction algorithms either leverage the inherent structure of the original data or depend on the modeling algorithm's capacity to extract the most influential spectral bands relevant to the task objective. With the continuous development of deep learning algorithms, both feature extraction and feature modeling are rapidly advancing. Nie et al. [19] constructed a deep convolutional neural network that surpasses the support vector machine (SVM) in the hybrid okra seed identification. Even when dealing with 35 barley seeds varieties, the convolutional neural network (CNN)-based model was able to keep its superior prediction accuracy [20]. Sohn et al. [21] blended the visible and NIR spectra of hybrid and transgenic seeds for classification using CNN and achieved a testing rate of 98.9%. Undoubtedly, the advent of deep learning algorithms has unlocked the potential to leverage complex spectral features for identification tasks. The increasing adoption of deep learning in spectral analysis not only enhances model inference efficiency but also enables the extraction of crucial bands based on deep learning models, thereby alleviating the burden of spectral data analysis. Methods that can tackle the hidden patterns inside deep learning models without reducing the performance are also preferred and beneficial to resolve the “black box” dilemma in deep learning.

In this paper, the NIR and terahertz spectra of rice seeds from multiple genotypes along with their transgenic version were collected and analyzed. A cascade pipeline method based on deep learning was developed to extract and utilize the essential spectral features for screening transgenic seeds in a precise and rapid way. The metabolic variability of implementing the same foreign gene *cry1Ab/cry1Ac* was studied to provide a detailed reference for the results derived from the discriminant models. By constructing a feature excavation algorithm based on the modified guided backpropagation algorithm, several crucial bands were able to be distilled to reduce the modeling expense and increase inference speed. The major objective of this work is to (a) explore the spectral variation regularity among different genetic background rice seeds, (b) fabricate cascade identification models based on deep learning to identify GM rice seeds, and (c) extract essential features in NIR and terahertz spectra to build a more concise model with promising performance.

## Materials and Methods

**Fig. 1.**
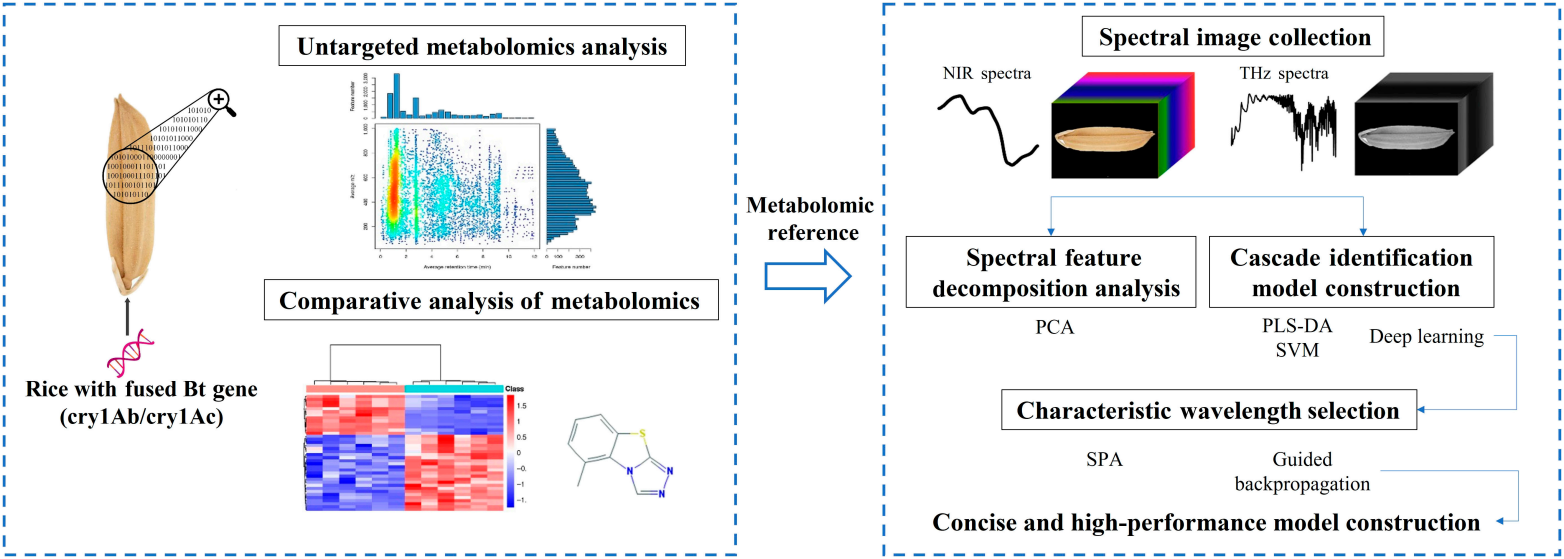
Flowchart of untargeted metabolomics analysis, spectral data acquisition, and data analysis for discriminating GM rice seeds.

The general components of the data processing in this study were shown in Fig. 1. First, the untargeted metabolomics analysis was followed by the comparative analysis of the results. This molecular composition evidence was introduced to verify the reliability of the identification models. Then, the NIR and terahertz spectral images of identical rice seeds were collected and processed before feeding into the feature decomposition model PCA and cascade models for variety and GM classification. The performance of selecting the essential wavelengths and frequencies was compared between the utilization of traditional dimensional reduction algorithms and proposed modified guided backpropagation. Finally, the concise, rapid, and reliable GM rice seed discrimination models were established to boost the advancement of GM product traceability.

### Sample preparation

**Fig. 2.**
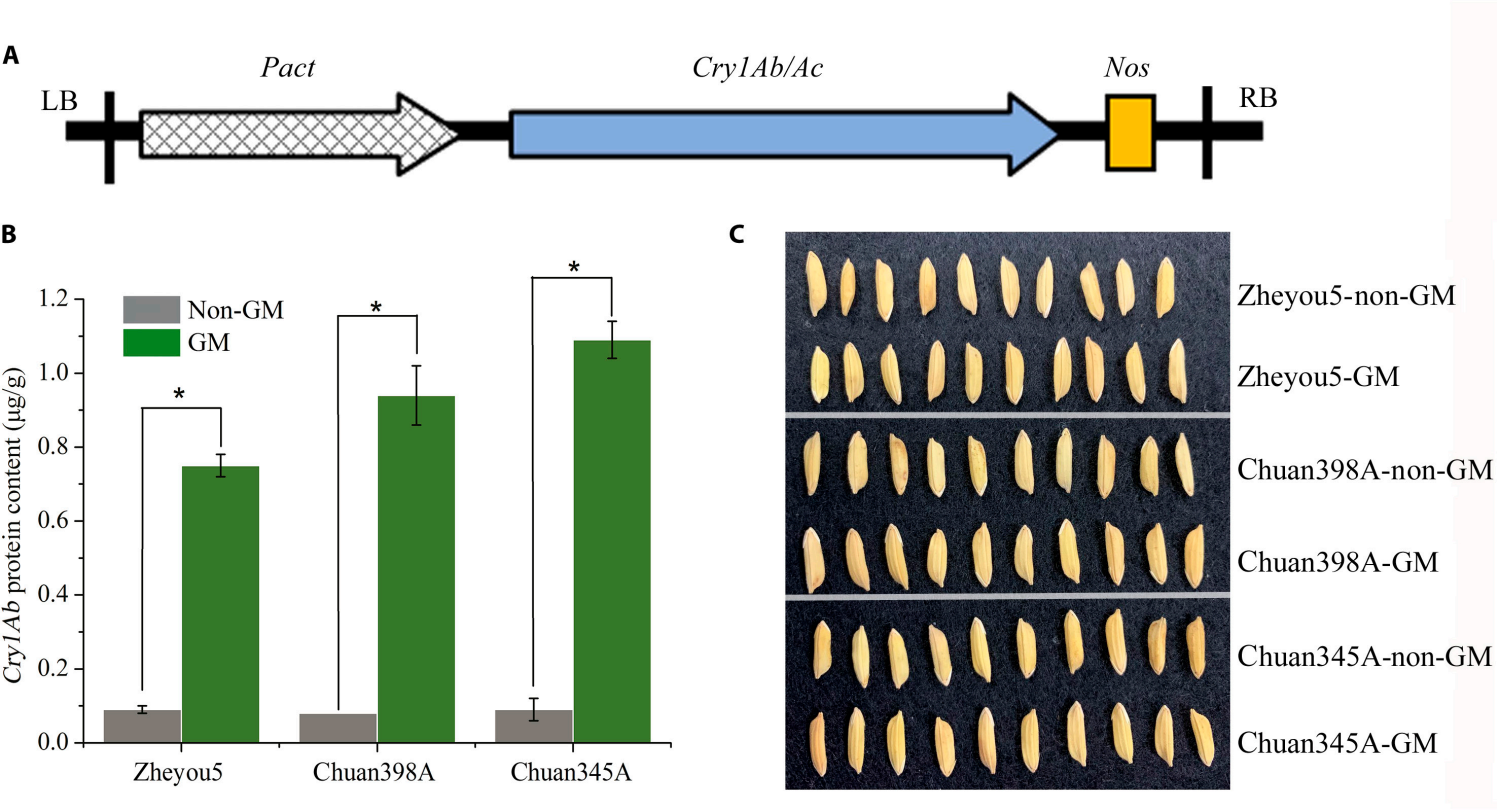
Material of transgenic *cry1Ab/cry1Ac* rice and their parent lines. (A) Structure of plant expression vector containing coding regions of *cry1Ab/cry1Ac* gene. (B) Enzyme-linked immunosorbent assay analysis results of *cry1Ab/cry1Ac* expression protein in rice seed. (C) Morphological traits of different rice seeds. LB, left border; Pact: Pact promoter; Nos: Nos terminator; RB, right border.

The GM rice seeds contained insecticidal tolerance *cry1Ab/cry1Ac* gene that originally came from Bt, and their unmodified control lines (non-GM) were provided by the Institute of Quality and Standard for Agro-Products, Zhejiang Academy of Agriculture Science, China. Three different genetic background rice varieties zheyou5, chuan389AX, and chuan345A were hosts for *cry1Ab/cry1Ac* gene from *Agrobacterium-tumefaciens*-mediated systems (Fig. 2A) and used as control lines. According to the literature, rice with fused Bt gene *Cry1Ab/Cry1Ac* is resistant against major target lepidopteran pests [22]. All the rice seeds were dried first by the plant breeder before putting into a paper bag for storage.

To measure the Bt-related protein content, we ground about 20 mg of the GM and non-GM rice seeds into a fine powder, respectively. Followed by the procedure instruction from the *Cry1Ab/Cry1Ac* plate kit (EnviroLogix Company, USA), Bt content was quantitatively measured. There were 3 replications for each sample and *Cry1Ab* calibrators. In the end, the results were tested by a plate reader at a wavelength of 450 nm. Standard curves were obtained using average values. Figure 2B showed that in non-GM rice lines, there were no or fewer *Cry1Ab/Cry1Ac* proteins compared with those in GM lines. Apparently, the level of the *Cry1Ab/Cry1Ac* protein content in the GM chuan345A was the highest among these 3 GM rice lines, and the protein content of GM chuan398A had a small distance to both GM zheyou5 and GM chuan345A. From the visual perspective, there were no obvious differences in morphological characters such as shape and perimeter in those rice seeds between their parental controls and the corresponding transgenic mutants (Fig. 2C).

### Metabolite profiling

The untargeted metabolomics of GM and non-GM rice seeds were analyzed using ultraperformance liquid chromatography-coupled mass spectrometry in negative and positive ion models by LC Sciences (Hangzhou, China). A total of 6 rice samples from every variety and transgenic status were randomly chosen. An ultraperformance liquid chromatography system (SCIEX, UK) performed the chromatographic separations. Metabolites eluted from the column were detected by a high-resolution tandem mass spectrometer TripleTOF5600plus (SCIEX, UK). Detail description of the metabolites profiling process could be found in [23]. The acquitted metabolomics files were processed and analyzed using the XCMS software (UC, Berkeley, CA, USA) in the R environment [24]. The online databases including Kyoto Encyclopedia of Genes and Genomes (http://www.kegg.jp/) and Human Metabolome Database (http://www.hmdb.ca/) were used to annotate and identify the level 1 and level 2 metabolites by matching the exact molecular mass data from samples with those from the database.

### Hyperspectral reflectance image collection

Hyperspectral images of 4,800 rice seeds in total were acquired in the NIR range of 874 to 1,734 nm with a band overall amount of 256 by the imaging system. Each genotype contained around 800 intact rice seeds. Two 150-W tungsten halogen lamps (Fiber-Lite DC950 Illuminator, Dolan Jenner Industries Inc., Boxborough, MA, USA) were applied for illumination in NIR spectral measurement system. An exhaustive description of the imaging system structure and relative components can be found in the previous study [15,25]. In the experiment, rice seeds on a black plate were placed on an electronically controlled conveyor belt for the hyperspectral line scan that can capture 320 point spectra at one time. The entire imaging process was optimized to ensure a duration of approximately 30 s, effectively minimizing the impact of water content variation. The distance between the sample and the hyperspectral camera was maintained at 130 mm, the belt speed was set to 15 mm/s, and the camera exposure time was adjusted to 4 ms. The image acquisition time was controlled to be around 30 s to ensure that there was no significant change in the seed moisture content. Because of the low signal-to-noise ratio engendered by the spectral camera and environment illumination, spectral features at the beginning range, as well as the end range, were discarded leaving 200 bands in the range of 958 to 1,630 nm. It is necessary to calibrate the raw hyperspectral image to highlight the reflectance signal from the sample itself. Therefore, a black reference that was produced by covering the camera and a white reference that came from capturing the reflectance of the white Teflon board were introduced. The mathematical formulas for calibration can be retrieved in [26].

### Terahertz absorbance image collection

The terahertz absorbance imaging was carried out on a terahertz time-domain spectrometer CCT-1800 in the range of 0.1 to 5 THz (China Communication Technology Co. Ltd, Shenzhen, China), which has been detailed described in [25]. A 780-nm femtosecond laser (Menlo Systems, Germany) was used to excite semiconductor devices to generate and receive terahertz signals. Only a section of the terahertz frequency that was 0.3 to 2.0 THz (93 bands) was chosen to be applied in the subsequent data analysis for the sake of reducing signal noise. Before moving rice seeds into the apparatus, they were supposed to be put into the oven to dry at 45 °C for 48 h to eliminate the strong absorption of water. Then, at each detection protocol, around 40 rice seeds could be dispersedly stuck on the transparent tape in the sample chamber of the transmission imaging module. The whole image collection process was conducted in a finely controlled environment in which the temperature was set to 20 °C and constant nitrogen gas was supplied to remove moisture in the air. Finally, the terahertz absorbance image with the field size of 50 mm × 50 mm was acquired through the supporting software. There were 3,378 rice seeds selected for terahertz absorbance image acquisition, which included 550 zheyou5-non-GM samples, 554 zheyou5-GM samples, 588 chuan398A-non-GM samples, 543 chuan398A-GM samples, 578 chuan345A-non-GM samples, and 565 chuan345A-GM samples.

### Spectral image preprocessing

Since plenty of rice seeds were scanned in a single spectral image, it was vital to use an image processing algorithm to separately extract the rice spectra first. In this study, the average spectra of each rice seed in its individual region were calculated using an adaptive threshold background segmentation [27] coupled with the connected components algorithm [28]. The spectral images at 1,363 nm were selected as the reference for segmenting rice seeds and obtaining their masks. The adaptive mean values, calculated within a moving neighborhood block with a size of 11, were used as the corresponding segmentation threshold. Following this, the connected component algorithm labeled each rice seed with a unique number, enabling the derivation of averaged spectra for each seed. Moreover, smoothing and baseline correction is an essential step to reduce noise, underline spectral peaks, and eliminate apparatus shift, which is especially beneficial to chemometrics modeling [29,30]. The moving average smooth filter with a window size of 5 was applied to remove outliers for both NIR spectra and terahertz spectra. In addition, baseline shift of terahertz spectral was subsequently corrected by the adaptive iteratively reweighted penalized least-squares algorithm with a penalty coefficient of 1 and 15 iterations round. The algorithm can alter the weights of sum of squares errors between the signal and baseline [31].

### Data analysis methods

#### Spectral feature decomposition methods

The PCA algorithm is able to project the high-dimensional features to low-dimensional space in an unsupervised way and output the most representative features called PCs [32]. The first few PCs explain the greatest amount of information inside the spectral data. So, it becomes accessible to distinguish and cluster the different samples from the PCA score plot. The average spectrum data of GM and non-GM rice seeds were fed into the PCA to reduce the hundreds of bands into 2 main PCs for direct clustering scatter visualization. The first 2 PCs that explained the most variance of the original spectra gave out the fundamental knowledge of the basic structure of involved spectral data and the difficulty of direct analysis without the help of a supervised learning algorithm during the process of GM rice seed identification.

#### Benchmark discriminant methods

Benefiting from the high performance and low computation cost, PLS-DA is commonly treated as a benchmark in spectral feature classification [33,34]. PLS-DA combines the advantages of PCA and partial least-squares methods and can efficiently deal with the problem of multicollinearity. In this study, spectra from different rice varieties and transgenic status were set up to be the independent variable. On the basis of preexperimental results, the number of hidden component variables was set to 5 to ensure good interpretability. In the inference section, corresponding output labels were encoded in the form of one-hot. Accordingly, the index of maximum value from the output units was determined as the final discriminant result.

SVM is a powerful classification algorithm built on the empirical risk minimization principle [35]. It can not only process linear and nonlinear data but also maintain stable generalization performance while dealing with fewer features. By virtue of introducing the radial basis function that was chosen to be the kernel function in this paper, the high-dimensional feature turned out to be more operable. Considering that SVM was mainly designed for binary classification, the one-versus-rest strategy was applied to carry out the multiclassification task. The 10-fold cross-validation was conducted to get the final discriminant results for both PLS-DA and SVM. The regularization parameter in SVM controls the misclassification tolerance and decision boundary complexity. The decision margin becomes wider when the regularization parameter is set to a smaller value. As for the label encoding, a sequential number from 0 to the maximum class amount is preferred. Apart from cross-validation, for all the discriminant methods, the original dataset was split in a ratio of 7:3 to construct the training dataset and testing dataset.

#### Characteristic wavelength selection methods

Although continuous and narrow wavelengths in spectra bring rich sample information, it also gives rise to irrelevant and redundant features that will decrease the inference speed of the model and increase the risk of overfitting [36]. Selecting characteristic wavelengths to build concise and rapid pipeline methods for transgenic rice seed classification is essential. SPA is able to single out representative features in iterative vector projections. By comparing and reserving the projected vector with the longest length, a series of wavelength candidate subsets are formed and fed into the PLS-DA model to compare their accuracy in cross-validation. Finally, only one subset with the highest accuracy and limited elements was kept. Here, the maximum selection number for wavelength was set to 13. Detailed information regarding the selected number of wavelengths for variety classification and GM status determination can be found in Figs. S1 and S2, respectively.

**Fig. 3.**
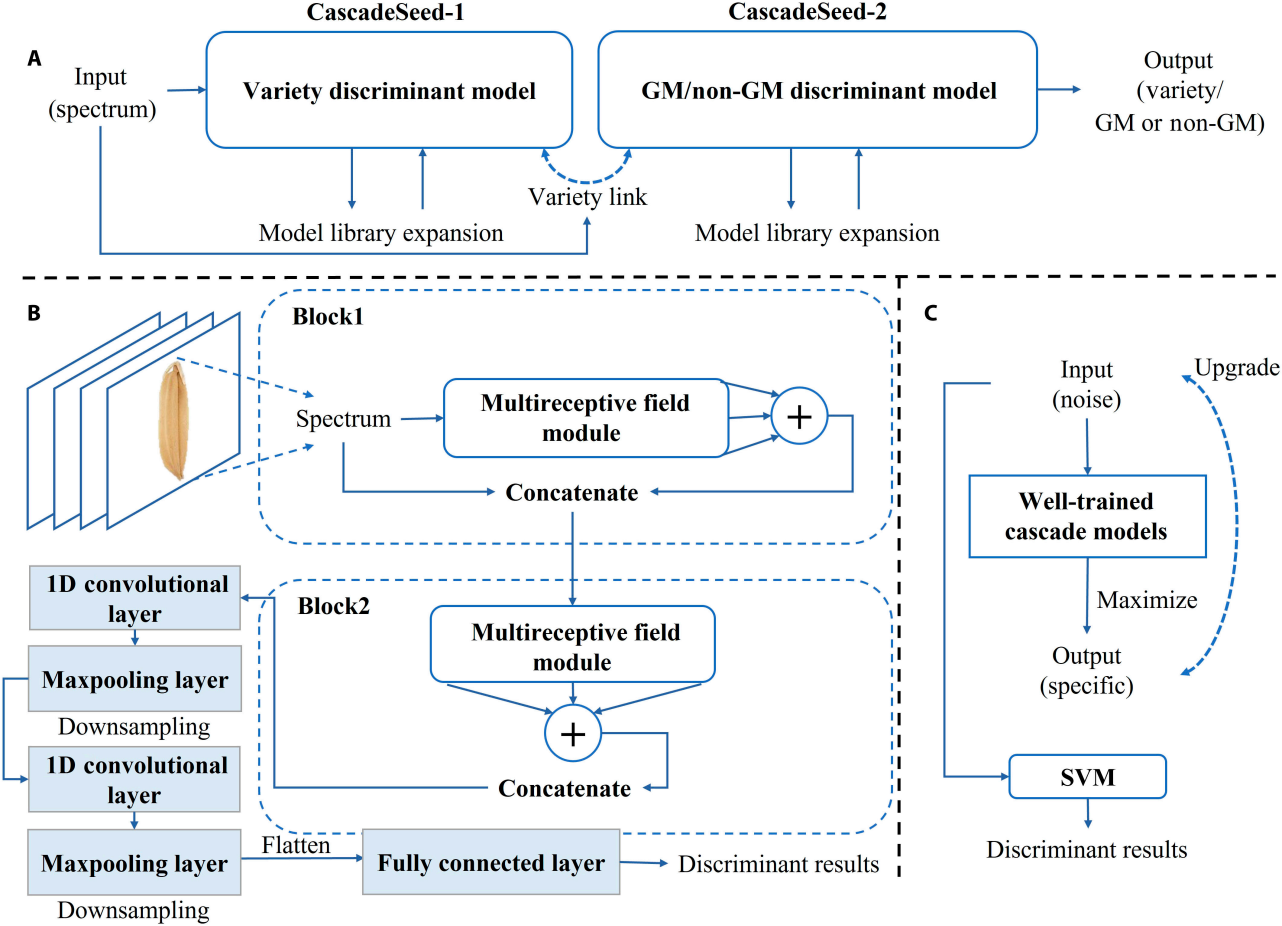
The basic structure of proposed cascade models for screening transgenic rice seed. (A) Detailed mechanism diagram inside the inference phases. (B) Comprehensive module composition of CascadeSeed-1 and CascadeSeed-2. (C) The proposed modified guided backpropagation algorithm for selecting features from cascade models.

As mentioned in the earlier study, the changing magnitude of rice seed spectra from different varieties was likely huge, which could overwhelm the slight variation inside the transgenic status of the same rice variety [25]. In addition, it might not be an appropriate method to directly identify the GM rice seeds without taking the variety factor into account. So, this paper proposed a cascade discriminant method consisting of a rice variety discriminant model called CascadeSeed-1 and a transgenic status discriminant model called CascadeSeed-2 to take specific action in each identification phase as shown in Fig. 3A. In the first phase, CascadeSeed-1 would predict the rice variety given the input spectra. In the second phase, specialized CascadeSeed-2 took the identical spectra to screen the GM rice seed in the same variety to output the final transgenic status. In addition, this kind of pipeline method provided an interface for expanding the model library that can boost the models’ generalization ability for unknown rice varieties.

Several classic and practical deep learning modules including the convolutional neural network module and fully connected network module were exploited to construct the CascadeSeed-1 and CascadeSeed-2. Notably, as Fig. 3B illustrated, these 2 models shared the same architecture and dissimilar structural parameters. Initially, a multirespective field module took the responsibility to process the average spectrum. Table 1 gave an elaborate introduction to the module’s structural hyperparameters. There were 4 one-dimensional (1D) convolutional layers to extract features from seed spectra. In comparison with 2D convolutional layers, 1D convolutional layers were more proper to bring out the property of sparse interactions and parameter sharing in the process of spectral vector classification. Moreover, 1D convolutional layers with fewer trainable parameters would reduce the trend of overfitting in a relative dataset. The other unequivocal attribute of the multirespective field module was the increasement in the size of the respective field with different kernel sizes, which hopefully could let the model learn more scalable features. In successive steps, alongside the channel axis, the sum map of multirespective fields was concatenated with the original spectrum to let the model reuse the former features while building a deeper architecture. There were 2 connected blocks involved with a similar data processing procedure before the derived features were transferred into the common 1D convolutional layers and 1D max-pooling layers for distillation. The number of filters in these 2 1D convolutional layers with a stride of 1 was set to 64 and 32, respectively. Both the 1D max-pooling layers with a stride of 2 and 1D convolutional layers had a kernel size of 3 and a padding size of 1. The rectified linear unit (ReLu) activation function that can deal with the influence of the gradient disappearance problem was introduced in-between layers to enhance the nonlinearity of the network. At the end part of the model, concerning feature sizes from the convolutional module, fully connected layers automatically created an intensive link between the flattened feature and the output results.

**Table 1.** Structural hyperparameters of the multireceptive field modules.

Module index	Layer name	Hyperparameters	Input size	Output size
1	Conv1d-1	(128, 1, 1, 0)^a^	(256, 1, 200)^b^	(256, 128, 200)^b^
	Conv1d-2	(128, 3, 1, 1)^a^	(256, 1, 200)^b^	(256, 128, 200)^b^
	Conv1d-3	(128, 5, 1, 2)^a^	(256, 1, 200)^b^	(256, 128, 200)^b^
	Conv1d-4	(128, 7, 1, 3)^a^	(256, 1, 200)^b^	(256, 128, 200)^b^
2	Conv1d-5	(64, 1, 1, 0)^a^	(256, 129, 200)^b^	(256, 64, 200)^b^
	Conv1d-6	(64, 3, 1, 1)^a^	(256, 129, 200)^b^	(256, 64, 200)^b^
	Conv1d-7	(64, 5, 1, 2)^a^	(256, 129, 200)^b^	(256, 64, 200)^b^
	Conv1d-8	(64, 7, 1, 3)^a^	(256, 129, 200)^b^	(256, 64, 200)^b^

^a^Kernel amount, kernel size, stride size, and padding size.

^b^Batch size, channel amount, and spatial size.

Similarly, the model output was encoded as a numeric vector with the same size as the number of sample categories that was 3 for CascadeSeed-1 and 2 for CascadeSeed-2. Besides, the softmax function as the last activation function was performed to convert the output into a probability distribution for calculating the confidence score. Losses representing the distribution distance between the true label and predicted value were quantized by the CrossEntropyLoss function (Eq. 1) to guide the parameter update. The loss optimization method based on stochastic gradient descent in this study was determined to be the Adam algorithm with a learning rate of 0.001 [37]. In addition, Kaiming normalization was used to initialize all weights inside the model for better convergence [38]. In the course of 10,000 epoch training, the accuracy of the validation set that constitutes 10% of the training set was treated as the reference to save the model’s parameters.

$$P_{\mathrm{ij}}=\frac{e^{z_{\mathrm{ij}}}}{\sum_{j=1}^{N}e^{z_{\mathrm{ij}}}}\;\text{for}\;j=1,2,\cdots N$$ (1)

where *z_ij_* represents the output of the last fully connected layer for the sample *i* in class *j* and *P* represents the classification possibility of each sample.

As shown in Fig. 2C, a modified guided backpropagation algorithm was proposed to extract the interior learned knowledge from well-trained cascade models. To start with, we treated a fixed random noise vector with the same size of input spectra from a uniform distribution as a new input. Then, the noise vector would be updated on the basis of a novel loss function to extract information about the knowledge learned by the established model. The objective loss function comprised 3 components, as shown in Eq. 2. In the first components, the predicted output value of a prespecific class provided a negative direction so that the noise vector could be reconstituted to class-specific spectra that would contain extensive information for wavelength selection. Then, the values of the other neurons were treated in a positive direction to prevent the noise from learning their pattern. In the last part, the penalty ability of the LASSO function forced the feature inside the noise to become sparser; therefore, only significant wavelength would be highlighted [39]. Stochastic gradient descent optimizer coupled with a learning rate of 6 was applied to perform 15,000 parameter iterations. In this manner, every predicted class from CascadeSeed-1 and CascadeSeed-2 could acquire their identity spectra for characteristic wavelength selection.

$$L_{C}=-f_{C}(x)+\sum_{i=1}^{C-1}f_{i}(x)+\sum_{j=1}^{N}x_{j}\;\text{for}\;j=1,2,\cdots N$$ (2)

where *f_C_*(*x*) and *f_i_*(*x*) represent the final neuron values of *C* class and non-*C* with input noise *x* and *j* denotes the index of the wavelength of the input noise.

### Analytical metrics and relative tools

For the untargeted metabolomic analysis, one-way analysis of variance (ANOVA) combined with Tukey’s range test was performed to examine metabolic discrepancies between GM and non-GM rice seeds. Differences at the level *P* ≤ 0.05 were deemed statistically significant. To evaluate the metabolomics profiles of rice seed from GM traits, we conducted a hierarchical cluster analysis based on the top 15 largest metabolic difference between each non-GM and GM rice variety after normalization. The metrics for evaluating the discriminant models included accuracy, macro F_1_ score that evaluated each class equally, and micro F_1_ score that evaluated each observation equally. Utilizing these metrics as indicator, both the performance of the model and the unbalanced sampling situation could be precisely detected.

$$\text{Precision}=\frac{\mathrm{TP}}{\mathrm{TP}+\mathrm{FP}}$$ (3)

$$\text{Recall}=\frac{\mathrm{TP}}{\mathrm{TP}+\mathrm{FN}}$$ (4)

$$\text{Macro}\;F_{1}=\frac{\sum_{i=1}^{N}\frac{2\times {\text{Precision}}_{i}\times {\text{Recall}}_{i}}{{\text{Precision}}_{i}+{\text{Recall}}_{i}}}{N}$$ (5)

$$\text{Micro}\;F_{1}=\frac{2\times \sum_{i=1}^{N}\frac{{\mathrm{TP}}_{i}}{{\mathrm{TP}}_{i}+{\mathrm{FP}}_{i}}\times \sum_{i=1}^{N}\frac{{\mathrm{TP}}_{i}}{{\mathrm{TP}}_{i}+{\mathrm{FN}}_{i}}}{\sum_{i=1}^{N}\frac{{\mathrm{TP}}_{i}}{{\mathrm{TP}}_{i}+{\mathrm{FP}}_{i}}+\sum_{i=1}^{N}\frac{{\mathrm{TP}}_{i}}{{\mathrm{TP}}_{i}+{\mathrm{FN}}_{i}}}$$ (6)

where TP, FP, and FN respectively mean the true-positive, the false-positive, and false-negative classification results and *N* is the number of discriminant classes.

The experiments were run using Python 3.7.13 (https://www.python.org/) with Jupyter Notebook to analyze hierarchical clustering, extract spectral data, and establish traditional discriminant models. In particular, the open-source framework PyTorch 1.12.1 (https://pytorch.org/) along with CUDA 11.3 was chosen to build and train those deep learning models. The Windows 10 operating system with AMD Ryzen 7 5800H central processing unit and NVIDIA GeForce (R) RTX 3060 graphics processing unit carried out all the data analysis and figure drafting.

## Results

### Metabolome analysis of transgenic rice lines expressing *cry1Ab/cry1Ac* gene

**Fig. 4.**
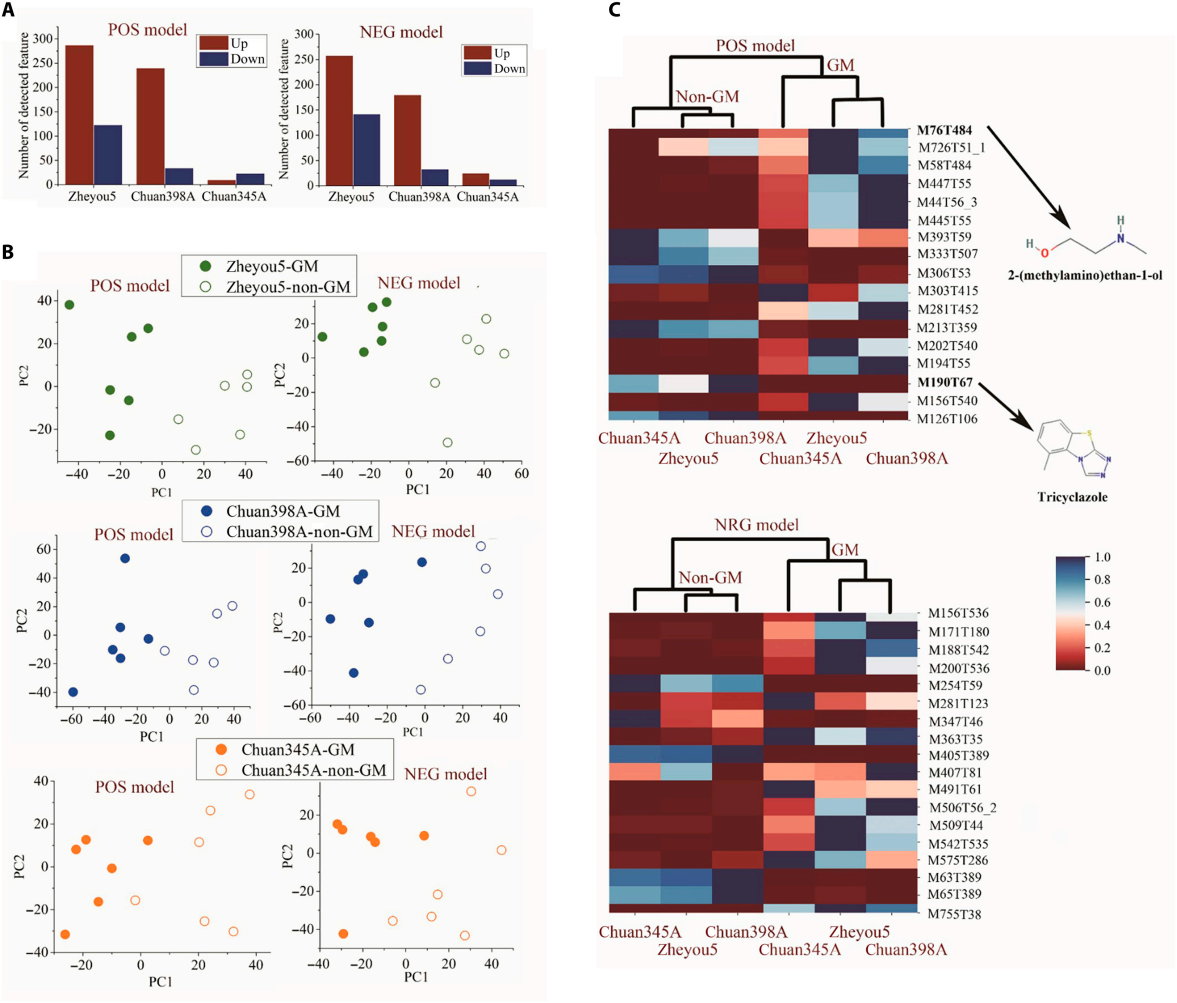
Untargeted metabolite profiling results of positive and negative models for GM and non-GM rice seeds. (A) Statistical histogram for different metabolites. (B) PCA scatter maps of metabolomes. (C) Heatmaps of the relative top 15 largest metabolic differences between the GM and non-GM rice seeds. The color bar, from red to blue, shows increasing levels of metabolite concentrations. POS, positive; NEG, negative.

Typically, risk assessment of GM products depends on the substantial equivalence comparison with their conventional counterparts. The intended and unintended GM effects with these different genotypic background rice seeds were evaluated by an untargeted metabolomics platform. The statistics of metabolites in Data S1 revealed that about 10,678 and 11,845 metabolites were acquired by the positive and negative models, respectively, 5,184 and 5,354 of which were annotated by level 1 and level 2 mass spectral data. Most of these metabolites were assigned to organic acids and their derivatives, lipids, lipid-like molecules, organ heterocyclic compounds, and organic acids and their derivatives (Figs. S3 and S4). In addition, significant differences were detected between GM and non-GM rice varieties. Figure 4A illustrated that being altered by the identical gene, rice variety zheyou5 obtained the greatest number of metabolites up-regulated and down-regulated in both positive and negative models. PCA performed on all the identified metabolites showed separations between GM and non-GM for all the rice varieties (Fig. 4B). Furthermore, it could be seen in Fig. 4C that the heatmaps constructed by the concentration of the top 15 distinct metabolites between GM and non-GM gave out the overall metabolic changing pattern. A clear connection between the expression of Bt proteins in rice seeds and alterations in the concentration of metabolites was detected. Moreover, similar variations were found among the different genetic background rice samples related to the same genetic engineering. It was also apparent that in the terms of metabolic composition, zheyou5 was more resemble chuan398A than chuan345A, which could cause some confusion for models based on information of the composition. All in all, the seed metabolic compounds provided solid evidence to verify the feasibility and reliability of the spectral methodology.

### The GM rice seed discrimination based on cascade models

#### Spectral feature variation pattern

**Fig. 5.**
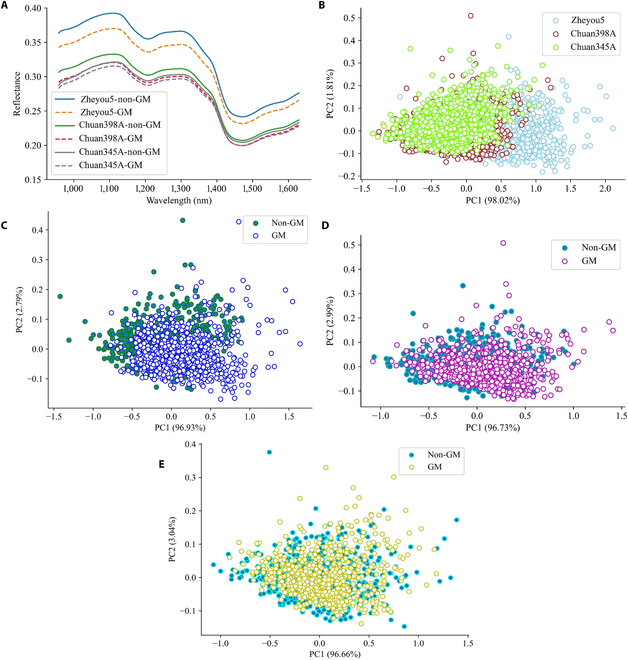
Spectral analysis results of GM and non-GM rice seeds based on NIR spectra. (A) Average spectrums from all rice seed varieties. (B) PCA scatter maps in the terms of variant seed variety. (C to E) PCA scatter maps in the terms of GM status for zheyou5, chuan398A, and chuan345A, respectively.

Since the spectral measurement tool can nondestructively retrieve the constitutional features of tested samples, it was conceivable to manage GM seed detection, while no information about modified gene sequences was available. In Fig. 5A, the spectral curve patterns and trends among genotypes were found to be similar. However, noticeable shifts were also observed in the reflectance values. It could be found that the reflectance value of the non-GM rice seed appeared to be greater than that of its GM counterpart. Furthermore, there was a significant magnitude of spectral variation observed among these varieties, which provided compelling evidence for the importance of developing cascade identification methods. The results obtained from dimensional decomposition PCA were summarized in Fig. 5B to E. Large overlapping areas could be observed in Fig. 5B, indicating the difficulty of relying solely on the original data structure of the NIR spectra. It is imperative and necessary to extract the hidden spectral features to achieve promising classification results.

**Fig. 6.**
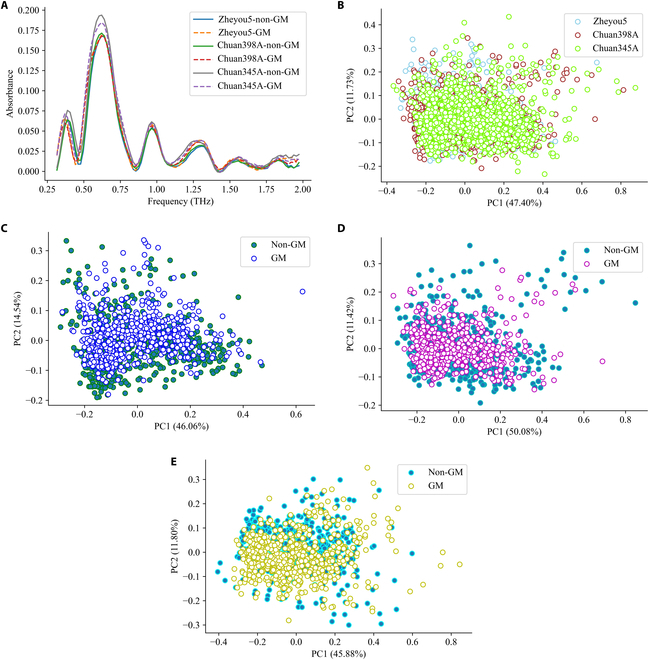
Spectral analysis results of GM and non-GM rice seeds based on terahertz spectra. (A) Average spectrums from all rice seed varieties after interval selection. (B) PCA scatter maps in the terms of variant seed variety. (C to E) PCA scatter maps in the terms of GM status for zheyou5, chuan398A, and chuan345A, respectively.

After baseline correction and noise smoothing, the unambiguous shape of terahertz spectra was manifested, and diverse intravariety and intervariety curve changes were captured as well in Fig. 6A. In comparison with NIR spectra, the visual separability of terahertz spectra from different genotypes and GM status was relatively poor. There were several observable absorption peaks at around 0.3, 0.6, 1.0, and 1.25 THz. In the range of 0.5 to 0.75 THz, the highest terahertz absorption values were collected in chuan345A, which further pointed out the difference between these genotypes. The data distribution of spectral components (Fig. 6B to E)) was comparable to the results derived from NIR spectra. However, the proportion rates of PCs in terahertz-based scatter maps were much lower, which elaborated less inherent collinearity in the terahertz spectra [40]. In general, the original spectral features in terahertz exhibited greater potential than NIR in the primary exploration of variety classification and GM status identification.

#### Discriminant results of rice seed variety and GM status

The variety discriminant results regardless of inner genetic background based on NIR reflectance spectra and terahertz absorption spectra were presented in Table 2. As the first component of the cascade model, the CascadeSeed-1 was put to compete with other classic machine learning models. In terms of NIR spectra, PLS-DA achieved a training accuracy of 79.17% and a testing accuracy of 77.98%. SVM and CascadeSeed-1 outperformed PLS-DA with an accuracy of over 80%, while CascadeSeed-1 achieved the highest testing accuracy of 87.80%. None of these 3 models based on NIR spectra showed signs of overfitting. However, when using terahertz spectra as the data source, the performance disparity between these 3 models increased. The PLS-DA model acquired the lowest identification performance, while SVM achieved a far better performance with a testing accuracy of 89.45%. Nevertheless, there seemed to be an occurrence of overfitting during the training of SVM. CascadeSeed-1 based on terahertz spectra still obtained the best variety discriminant performance with a testing accuracy of 97.04% and an F1 score of 0.97, showing its superiority in utilizing the hidden spectral features.

**Table 2.** Detailed discrimination results of the rice variety based on various models (values ​​in bold represent the relative best performance in the results).

Data source	Modeling algorithm	Parameters	Training accuracy (%)	Testing accuracy (%)	F1 score (macro)	F1 score (micro)
NIR spectra	PLS-DA	5^a^	79.17	77.98	0.77	0.78
	SVM	1 × 10^2b^	82.93	81.39	0.81	0.81
	CascadeSeed-1	(1 × 10^4^, 5 × 10^−4^)^c^	89.33	87.80	0.88	0.88
Terahertz spectra	PLS-DA	5^a^	64.30	61.83	0.62	0.62
	SVM	1 × 10^2b^	97.34	89.45	0.89	0.89
	CascadeSeed-1	(1 × 10^4^, 5 × 10^−4^)^c^	**98.94**	**97.04**	**0.97**	**0.97**

^a^The number of latent variables for calibration.

^b^The regularization parameter.

^c^The number of training epoch and learning rate for the optimizer.

**Fig. 7.**
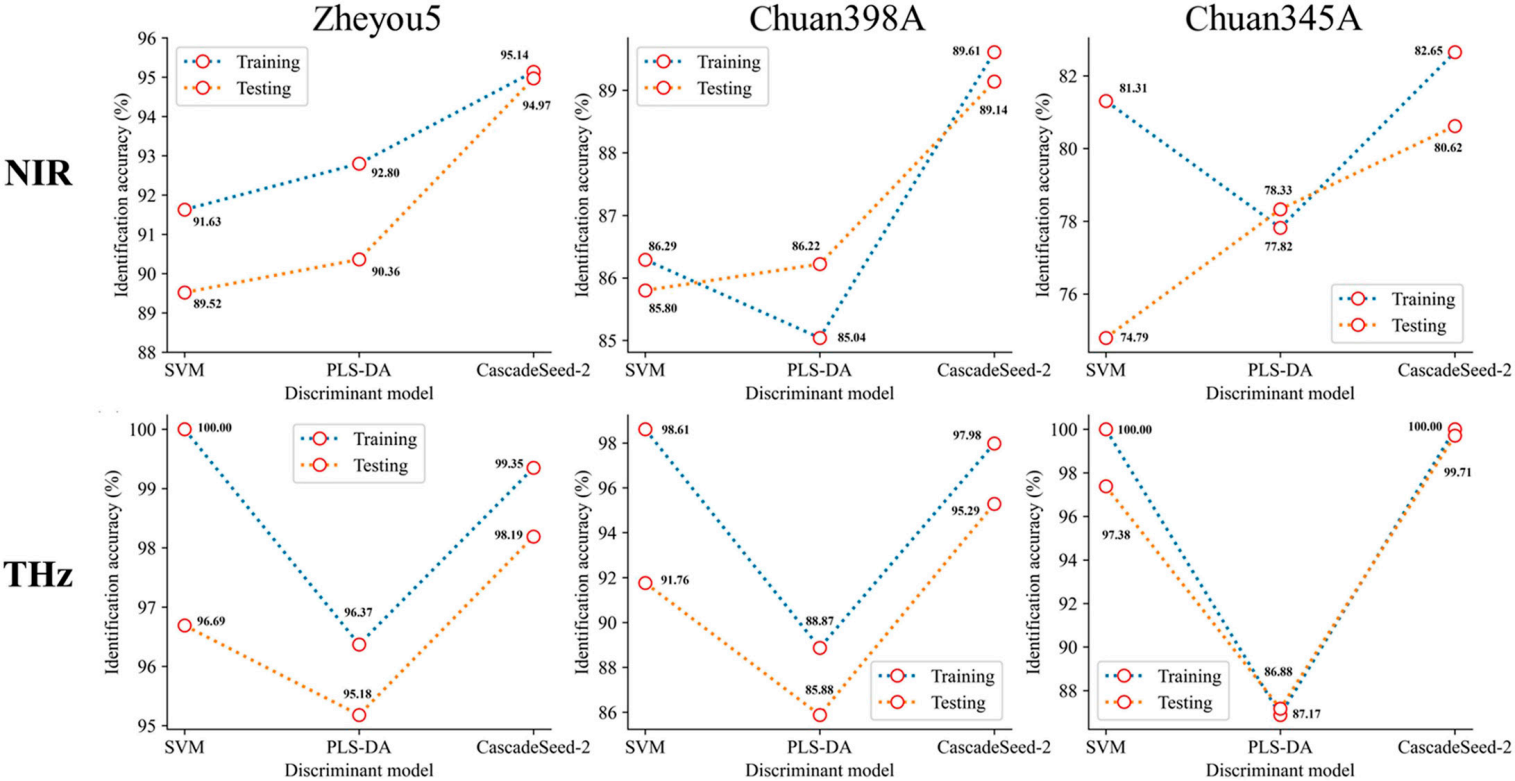
Comparison curves of classification accuracy based on various GM status discriminant models using NIR spectra and terahertz spectra.

In the second component of the cascade model, GM status identification was treated individually according to its basic genetic background. Figure 7 depicted the results obtained from 3 rice varieties and 2 spectral sources. In the process of identifying transgenic rice seeds in zheyou5, CascadeSeed-2 achieved an accuracy of 94.97% in the NIR testing set and an accuracy of 98.19% in the terahertz testing set. Despite SVM reaching nearly 100% training accuracy in terahertz spectra, CascadeSeed-2 consistently demonstrated stable performance from training to testing. Similar outcomes were observed in the identification of GM chuan398A and GM chuan345A. Among these 3 varieties, when using NIR spectra as the data source, GM zheyou5 exhibited the highest discriminant accuracy of approximately 94.97%, while the other 2 varieties achieved over 80% accuracy. For terahertz-based CascadeSeed-2, identification accuracies were consistently above 95%, with chuan398A showing the lowest performance. Overall, models established using terahertz spectra displayed higher discriminant capabilities compared to those based on NIR spectra, and CascadeSeed-2 exhibited a robust ability to identify transgenic seeds across different varieties.

### Identification of GM rice seed using wavelength selection methods

**Fig. 8.**
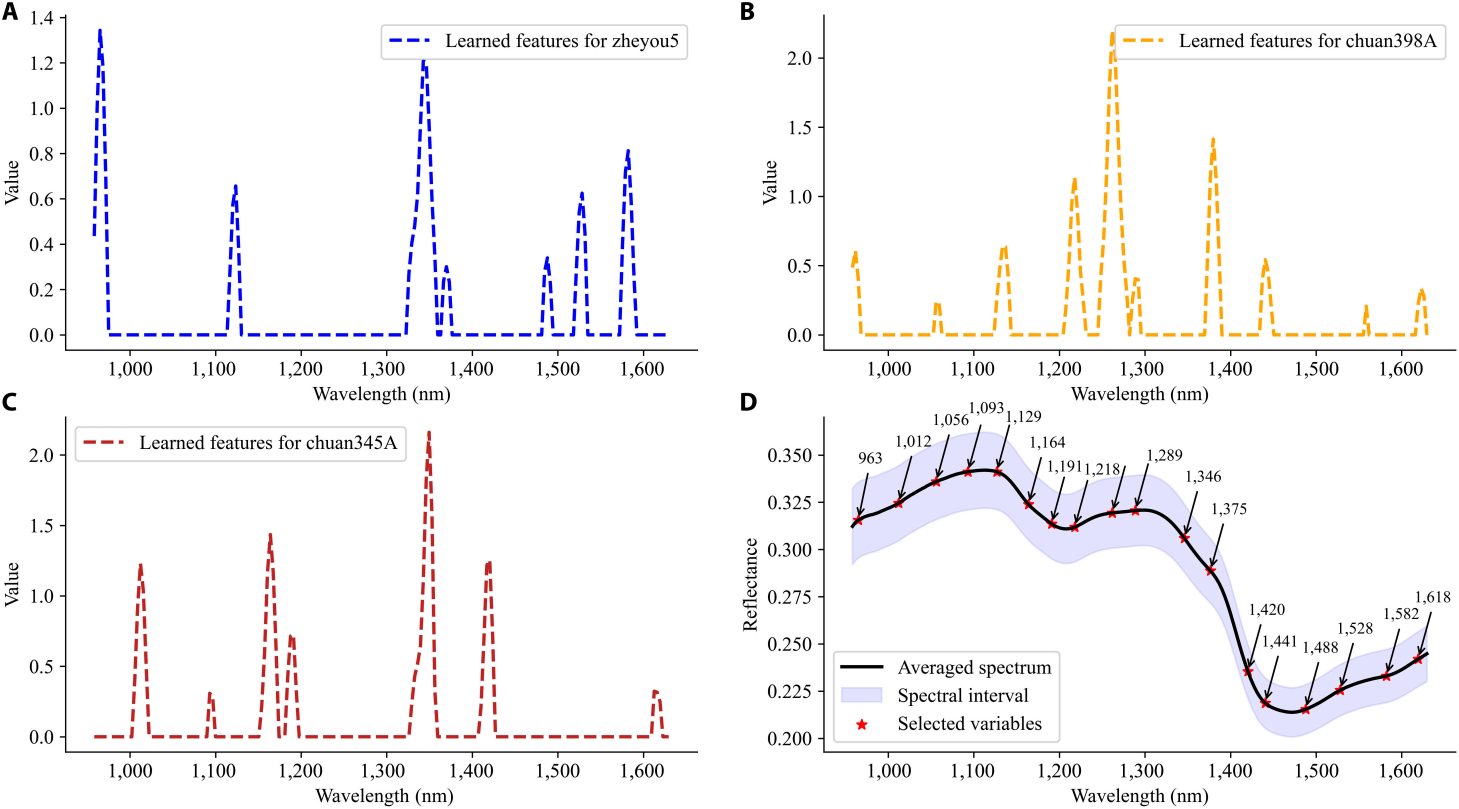
The NIR-related characteristics wavelength selection curves using modified guided backpropagation of CascadeSeed-1 for variety classification. (A to C) Learned identity features from zheyou5, chuan398A, and chuan345A, respectively. (D) Location of selected wavelengths on the average spectra.

**Fig. 9.**
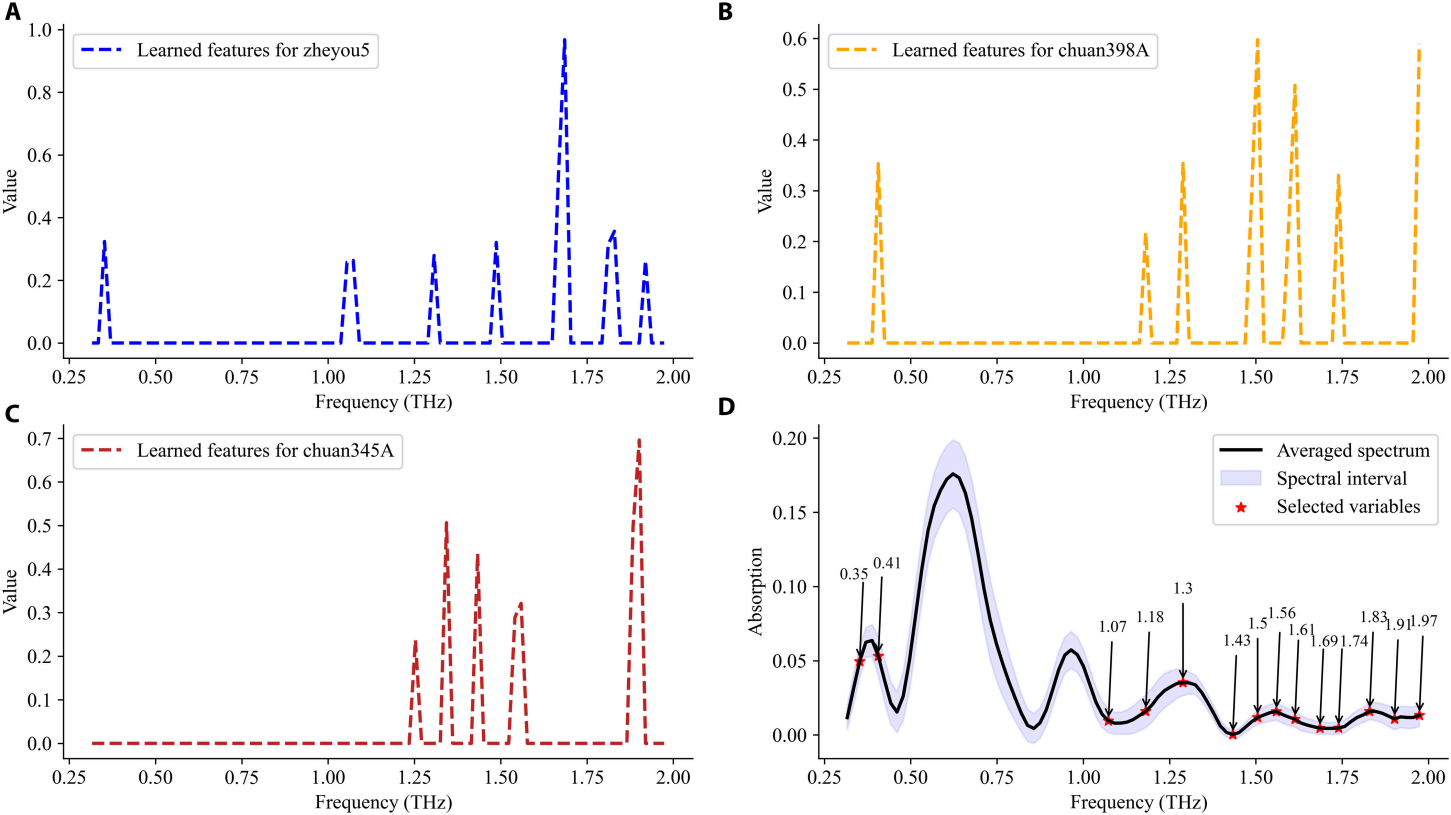
The terahertz-related characteristics wavelength selection curves using modified guided backpropagation of CascadeSeed-1 for variety classification. (A to C) Learned identity features from zheyou5, chuan398A, and chuan345A, respectively. (D) Location of selected wavelengths on the average spectra.

To construct a concise and efficient transgenic rice seed discrimination model, we eliminated redundant features presented in spectra using wavelength selection algorithms. The SVM model, known for its high performance and low computational cost, was chosen as the classifier to evaluate the proposed strategy. The characteristic wavelengths and frequencies selected by SPA and the proposed modified guided backpropagation for seed variety classification were summarized in Table 3. These selections could provide some insights into the differentiation of varieties based on chemical structures and component concentrations. In both NIR spectra and terahertz spectra, 7 distinct bands were uniformly identified within the observation range. However, the testing accuracies based on these spectral features extracted from SPA showed a degradation in performance, especially for the terahertz spectra, with accuracies of 81.05% and 68.34%, respectively. The guided backpropagation of CascadeSeed-1 singled out additional bands using the model’s parameters. Figures 8A to C and 9A to C depicted the extracted unique identity spectra for each variety, and the combination of all these selected wavelengths and frequencies was also presented in Table 3. It was evident that uniformity was maintained in the NIR spectra, while the majority of terahertz characteristic frequencies was concentrated in the range of 1.0 to 2.0 THz. Interestingly, the bands selected by SPA were completely covered by the modified guided backpropagation selection. Furthermore, despite a slight degradation in identification performance, acceptable and improved testing accuracies of 84.95% for NIR and 80.28% for terahertz were achieved, demonstrating the effectiveness of the proposed wavelength selection methods. The majority of CascadeSeed-1’s performance on rice variety classification was effectively preserved.

**Table 3.** Comprehensive discriminant accuracies of the seed variety based on SVM (*C* = 100) using selected characteristic wavelengths (values ​​in bold represent wavelengths that were discovered in both feature selection methods).

Data source	Wavelength selection algorithm	Selected wavelength (nm and THz)	Training accuracy (%)	Testing accuracy (%)
NIR spectrum	SPA	958, 1,019, 1,201, 1,339, 1,380, 1,410, and 1,562	83.74	81.05
	Guided backpropagation of CascadeSeed-1	**965**, **1,012**, 1,036, 1,057, 1,073, 1,093, 1,124, 1,171, **1,221**, 1,269, **1,346**, **1,417**, 1,441, 1,477, 1,528, **1,557**, 1,580, and 1,619	89.90	84.95
Terahertz spectrum	SPA	0.35, 0.59, 1.20, 1.49, 1.63, 1.90, and 1.97	73.39	68.34
	Guided backpropagation of CascadeSeed-1	**0.35**, 0.41, 1.06, **1.18**, 1.25, 1.30, 1.34, 1.43, **1.49**, 1.55, **1.60**, 1.68, 1.74, 1.82, 1.90, and **1.97**	91.62	80.28

Considering the promising capability of CascadeSeed-2 in GM status identification, a similar strategy was used for the variety-specific models to distill the essential bands for simplifying the discriminant process. The identification accuracies based on those the selected wavelengths and frequencies (Table 4) all suffered a decline compared with results in Fig. 7. For instance, the highest GM status identification rate of chuan398A based on SPA and NIR was still lower than that derived from the whole band modeling. However, given the marginal decrease in performance, it also provided evidence that the model’s fundamental discriminative abilities had been preserved. The detailed identity spectra for each variety derived from CascadeSeed-2 were presented in Figs. S5 and S6. Table 5 summarized the locations of these identity bands for comparison. It was evident that testing accuracies based on modified guided backpropagation of CascadeSeed-2 were generally higher than those based on SPA. Despite not always achieving higher accuracy, comparable and advantageous results were observed for certain varieties, such as zheyou5 with NIR spectra and chuan398A with terahertz spectra. A large portion of the bands selected through SPA were still encompassed by the bands chosen through the modified guided backpropagation method. It was achievable to maintain a good GM status identification ability while refining the spectral bands for rapid and concise discrimination.

**Table 4.** Comprehensive discriminant accuracies of transgenic status based on SVM (*C* = 100) using selected NIR wavelengths (values ​​in bold represent wavelengths that were discovered in both feature selection methods).

Variety	Wavelength selection algorithm	Selected wavelength (nm)	Training accuracy (%)	Testing accuracy (%)
Zheyou5	SPA	1,144, 1,208, 1,292, and 1,400	82.36	81.34
	Guided backpropagation of CascadeSeed-2	989, 1,022, 1,049, 1,093, **1,154**, **1,221**, 1,269, 1,329, **1,397**, 1,491, and 1,565	94.87	93.29
Chuan398A	SPA	1,117, 1,201, 1,329, 1,410, 1,434, and 1,629	84.95	84.76
	Guided backpropagation of CascadeSeed-2	965, 1,093, **1,134**, **1,194**, **1,343**, **1,403**, 1,538, and **1,606**	84.68	84.55
Chuan345A	SPA	1,117, 1,201, 1,410, and 1,441	79.16	77.92
	Guided backpropagation of CascadeSeed-2	968, 1,026, 1,086, 1,140, **1,205**, 1,272, 1,366, **1,417**, **1,457**, 1,508, 1,565, and 1,616	83.63	81.46

**Table 5.** Comprehensive discriminant results of transgenic status based on SVM (*C* = 100) using selected terahertz wavelengths (values ​​in bold represent wavelengths that were discovered in both feature selection methods).

Variety	Wavelength selection algorithm	Selected wavelength (THz)	Training accuracy (%)	Testing accuracy (%)
Zheyou5	SPA	0.32, 0.53, 0.93, 1.49, 1.69, 1.83, and 1.94	98.83	95.18
	Guided backpropagation of CascadeSeed-2	**0.35**, **0.46**, 0.80, **0.91**, 1.04, 1.14, 1.21, 1.33, **1.51**, 1.56, 1.63, **1.69**, **1.83**, **1.92**, and 1.97	98.70	97.29
Chuan398A	SPA	0.33, 0.39, 1.36, 1.43, and 1.96	88.24	82.35
	Guided backpropagation of CascadeSeed-2	**0.33**, 0.80, 0.91, 1.13, 1.20, 1.31, **1.36**, **1.43**, 1.49, 1.56, 1.65, 1.74, 1.83, and **1.92**	90.64	87.35
Chuan345A	SPA	0.33, 0.39, 1.11, 1.18, 1.61, 1.74, 1.85, and 1.97	88.12	85.42
	Guided backpropagation of CascadeSeed-2	0.50, 0.64, 0.75, 0.80, 0.95, 1.04, **1.11**, **1.18**, 1.25, 1.34, 1.42, 1.47, 1.54, **1.60**, 1.69, **1.79**, **1.87**, and **1.96**	91.12	85.71

## Discussion

### The inner connections between metabolome analysis and spectral analysis

**Fig. 10.**
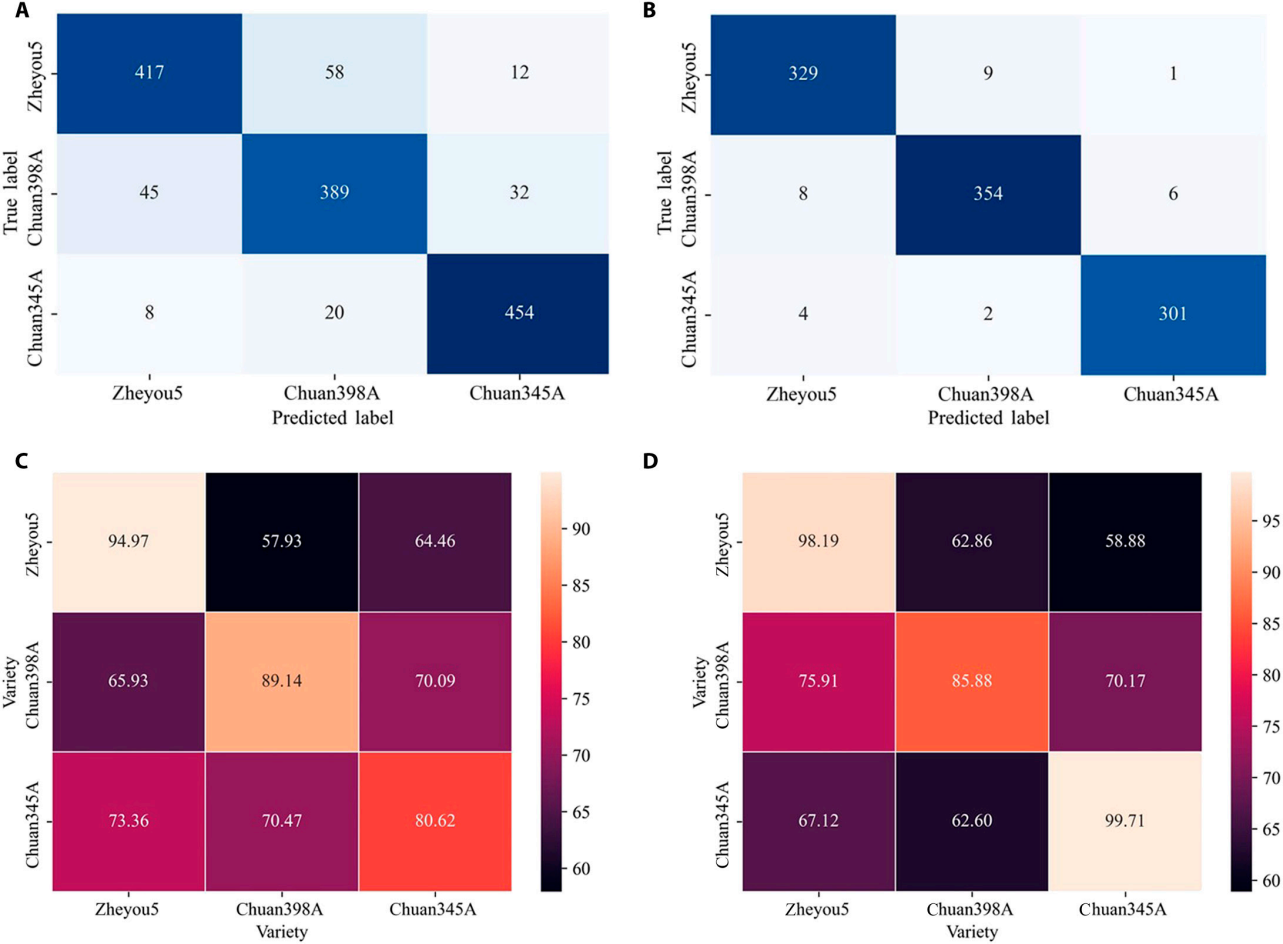
Confusion matrices of rice seed variety discrimination (A and B) and GM status cross-classification matrices derived from CascadeSeed-2 (C and D). (A) and (C) are the results based on NIR spectra, and (B) and (D) are the results based on terahertz spectra.

Results from metabolome analysis of these transgenic seeds depicted that because of the variant expression of introduced gene *cry1Ab/cry1Ac*, a series of organic compounds inside the rice seeds were altered without evidently affecting external appearance (Figs. 2C and 4). That provided solid evidence that there were inherent differences in the content of metabolic compounds among the studied samples. The spectral imaging technology was a perfect tool to quantically capture these interior substance variations. As presented in other literature, even the content of seed protein and starch could be quantified by utilizing these spectral features [11,41]. The terahertz spectra are quite capable of biomacromolecule content in the samples, which makes it a perfect tool to capture discriminant information [42]. Therefore, the results from the metabolome analysis served as a reliable reference for the discriminant outcomes. During the comparison of average spectra from the NIR range and terahertz range, distinct variation regularities with substantial information inside these rice seeds could be observed. More specifically, as shown in Fig. 10A and B, evident confusion between zheyou5 and chuan398A could be observed in both NIR-based model and terahertz-based model. At the same time, the metabolome analysis in Fig. 4A revealed a comparable number of detected features between these 2 genotypes. This parallel trend further confirmed that the extracted spectral features could reflect the metabolic content, thereby affecting the performance of the discriminant model. The accuracy matrices of cross predictions using the GM status identification model based on NIR and terahertz spectra were shown in Fig. 10C and D. It could be found that the performance of CascadeSeed-2 decreased in assessing other rice seed varieties, which could be attributable to the varied compound disparity degrees between GM and non-GM among these genotypes (Fig. 2B). The discernibility of the rice seed’s intrinsic composition diversity boosted the feasibility of applying the cascade model for transgenic identification.

### The performance of established cascade models

It was clear that the proposed cascade modeling approach attained a promising performance in accurately recognizing transgenic rice seeds from different genetic backgrounds by utilizing the powerful feature extraction ability of deep learning. The cascade modeling approach outperformed classic machine learning methods in the cascade task of seed variety classification and GM status identification with the highest accuracy of 97.04% and 99.71%, respectively. Some previous studies used deep learning algorithms combined with spectra features for seed-related classification. Coated maize varieties were identified with an accuracy of over 90% using the long short-term memory model combined with NIR spectra [43]. Huang et al. [44] proposed a lightweight convolutional neural network for detecting damaged soybean seeds and achieved 96.2% accuracy. Comparable identification accuracy could also be found in our proposed methods. However, the novel idea of the cascade modeling approach was revealed not only in the utilization of deep learning but also in the strategy of dividing the transgenic seed discrimination into 2 separate procedures. Separating the transgenic seed identification process with a cascade model avoided computation trouble and lowered the burden of inference. For some complicated object detection tasks, the effectiveness of the cascade model structure had already been proved that it could maximize the capability of the individual model to finally improve the general results [45,46].

### The effectiveness of modified guided propagation algorithm

The proposed guided backpropagation algorithm, based on the cascade model, outperformed the conventional SPA algorithm in selecting characteristic wavelengths, enabling the development of a concise and rapid discriminant method. In addition to the powerful feature extraction ability of deep learning, the well-designed loss function guided the backpropagation of input noise, which played a crucial role in identifying these wavelengths. Feng et al. [47] also used a guided feature extraction method, known as guided gradient-weighted class activation mapping, to select characteristic wavelengths for accurate rice blast disease detection. The effectiveness of spectral feature selection using the LASSO function has also been demonstrated [48]. However, these methods heavily relied on predesigned model structures, which may limit their applicability. Notably, the NIR wavelengths selected by the guided backpropagation of CascadeSeed-1 and CascadeSeed-2 not only covered the SPA range but also correlated with specific functional group stretching vibrations observed in the metabolic results. For instance, wavelengths around 995 nm represented the second overtone of N–H stretching, around 1,171 nm represented the second overtone of C–H stretching, around 1,346 nm corresponded to the first overtone of amide B, and around 1,517 nm represented the first overtone of O–H stretching [49]. The strong NIR absorption at 1,551 nm was associated with the N–H first overtone of protein changes [50]. The superior performance of terahertz spectra compared to NIR spectra could be attributed to the terahertz spectra of rice seeds preserving their strong ability to detect biomacromolecules [51]. Although this study did not precisely identify the compounds detected by terahertz, our results, validated by metabolic analysis, offer a reliable and innovative pipeline method to accelerate the inference speed of transgenic rice seed discrimination. Future research should include a wider range of rice seed varieties to expand the spectral dataset and enhance the screening capability for transgenic rice seeds. In addition, it is essential to establish an exact correspondence between metabolites and spectral features and analyze the interrelationships among the extracted feature bands to further simplify the model while enhancing its inference capability.

### Conclusion

In this study, a nondestructive, effective, and rapid pipeline method was proposed with respect to labeling and traceability requirements of GM organisms in the agro-food markets. Two spectroscopies including NIR hyperspectral imaging and terahertz imaging were involved to identify Bt-related GM rice seeds in 3 different genetic backgrounds. Initially, seed metabolome analysis revealed the biochemical and genetic background to changes in rice seeds as the function of the GM effect. Different GM statuses of all varieties were preliminarily captured in these average spectra features. Considering the variety variation in spectra, a cascade identification strategy was established to separately discriminate between the variety and corresponding GM status. The cascade models based on deep learning algorithms obtained a higher discrimination rate than the classic machine learning models indicating the superiority of the proposed methods. In the same genetic background condition, models built by terahertz spectra received a better discrimination ability. Moreover, a modified guided backpropagation algorithm successfully extracted characteristic wavelengths and frequencies reducing the cost of model construction. Although a slight decline in the identification accuracy could be observed after the band selection, the rapid inference speed would be beneficial to boost the real application of spectral technology. Our results demonstrated that the proposed transgenic rice seed discrimination pipeline method not only provided valuable and reliable prediction capabilities but also extracted essential spectral features potentially related to GM metabolic composition. It offers an alternative tool, coupled with novel algorithms, to enhance the development of rapid GM risk assessment.

## Data Availability

The data and relevant code supporting the findings of this study are available from the corresponding author upon request.

## Supplementary Materials

Supplementary 1 Fig. S1. The performance variation curves under different numbers of wavelengths selected by SPA in variety classification. Fig. S2. The performance variation curves under different numbers of wavelengths selected by SPA in GM status identification. Fig. S3. Metabolites detected and annotated in positive model according to Kyoto Encyclopedia of Genes and Genomes pathway classification. Fig. S4. Metabolites detected and annotated in negative model according to Kyoto Encyclopedia of Genes and Genomes pathway classification. Fig. S5. The NIR-related characteristics wavelength selection curves using guided backpropagation of CascadeSeed-2 for GM status identification. Fig. S6. The terahertz-related characteristics wavelength selection curves using guided backpropagation of CascadeSeed-2 for GM status identification. Data S1. Detail information of all metabolites detected in positive and negative model.
