# Supplementary material for: Concise Cascade Methods for Transgenic Rice Seed Discrimination using Spectral Phenotyping
Source: Plant Phenomics. 2023 Jul 28;5:0071. doi: 10.34133/plantphenomics.0071 (PMC10380542; doi:10.34133/plantphenomics.0071)
Supplement: Supplementary 1 — Fig. S1. The performance variation curves under different numbers of wavelengths selected by SPA in variety classification. Fig. S2. The performance variation curves under different numbers of wavelengths selected by SPA in GM status identification. Fig. S3. Metabolites detected and annotated in positive model according to Kyoto Encyclopedia of Genes and Genomes pathway classification. Fig. S4. Metabolites detected and annotated in negative model according to Kyoto Encyclopedia of Genes and Genomes pathway classification. Fig. S5. The NIR-related characteristics wavelength selection curves using guided backpropagation of CascadeSeed-2 for GM status identification. Fig. S6. The terahertz-related characteristics wavelength selection curves using guided backpropagation of CascadeSeed-2 for GM status identification. Data S1. Detail information of all metabolites detected in positive and negative model. [file plantphenomics.0071.f1.zip › SM.docx]

Supplementary Materials

Figure S1. The performance variation curves under different number of wavelengths selected by SPA in variety classification. (a) based on NIR spectra; (b) based on THz spectra.

Figure S2. The performance variation curves under different number of wavelengths selected by SPA in GM status identification. (a)-(c) the results of zheyou5, chuan398A and chuan345A based on NIR spectra; (d)-(e) the results of zheyou5, chuan398A and chuan345A based on THz spectra.

Figure S3. Metabolites detected and annotated in positive model according to KEGG pathway classification.

Figure S4. Metabolites detected and annotated in negative model according to KEGG pathway classification.

Figure S5. The NIR-related characteristics wavelength selection curves using guided backpropagation of CascadeSeed-2 for GM status identification. (a)-(c) learned identity features and their locations for zheyou5; (d)-(f) learned identity features and their locations for chuan398A; (g)-(i) learned identity features and their locations for chuan345A.

Figure S6. The THz-related characteristics wavelength selection curves using guided backpropagation of CascadeSeed-2 for GM status identification. (a)-(c) learned identity features and their locations for zheyou5; (d)-(f) learned identity features and their locations for chuan398A; (g)-(i) learned identity features and their locations for chuan345A.

Data S1. Detail information of all metabolites detected in positive and negative model.
