# Supplementary figures and images for: Concise Cascade Methods for Transgenic Rice Seed Discrimination using Spectral Phenotyping
Source: Plant Phenomics. 2023 Jul 28;5:0071. doi: 10.34133/plantphenomics.0071 (PMC10380542; doi:10.34133/plantphenomics.0071)

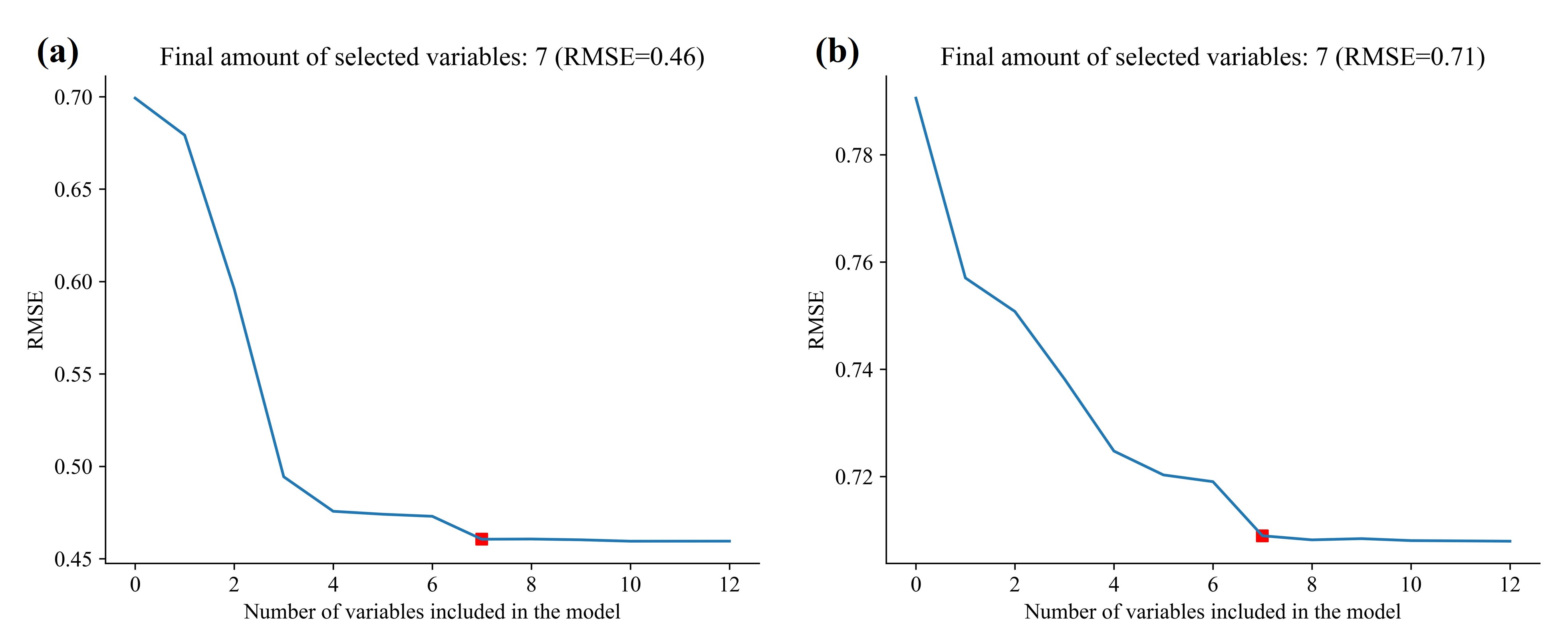

Supplement: Supplementary 1 — Fig. S1. The performance variation curves under different numbers of wavelengths selected by SPA in variety classification. Fig. S2. The performance variation curves under different numbers of wavelengths selected by SPA in GM status identification. Fig. S3. Metabolites detected and annotated in positive model according to Kyoto Encyclopedia of Genes and Genomes pathway classification. Fig. S4. Metabolites detected and annotated in negative model according to Kyoto Encyclopedia of Genes and Genomes pathway classification. Fig. S5. The NIR-related characteristics wavelength selection curves using guided backpropagation of CascadeSeed-2 for GM status identification. Fig. S6. The terahertz-related characteristics wavelength selection curves using guided backpropagation of CascadeSeed-2 for GM status identification. Data S1. Detail information of all metabolites detected in positive and negative model. [file plantphenomics.0071.f1.zip › Supplementary Figure S1.jpg]

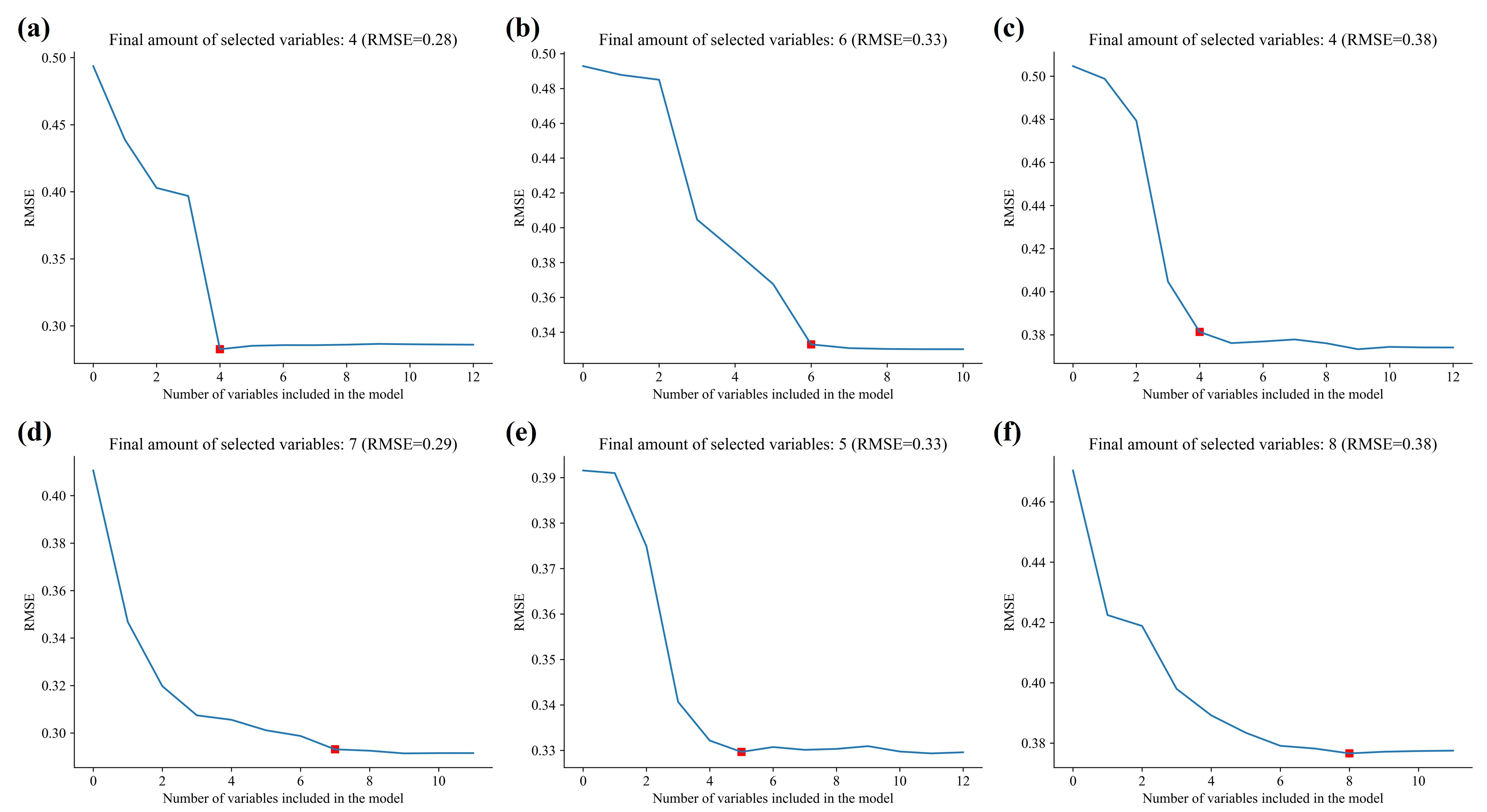

Supplement: Supplementary 1 — Fig. S1. The performance variation curves under different numbers of wavelengths selected by SPA in variety classification. Fig. S2. The performance variation curves under different numbers of wavelengths selected by SPA in GM status identification. Fig. S3. Metabolites detected and annotated in positive model according to Kyoto Encyclopedia of Genes and Genomes pathway classification. Fig. S4. Metabolites detected and annotated in negative model according to Kyoto Encyclopedia of Genes and Genomes pathway classification. Fig. S5. The NIR-related characteristics wavelength selection curves using guided backpropagation of CascadeSeed-2 for GM status identification. Fig. S6. The terahertz-related characteristics wavelength selection curves using guided backpropagation of CascadeSeed-2 for GM status identification. Data S1. Detail information of all metabolites detected in positive and negative model. [file plantphenomics.0071.f1.zip › Supplementary Figure S2.jpg]

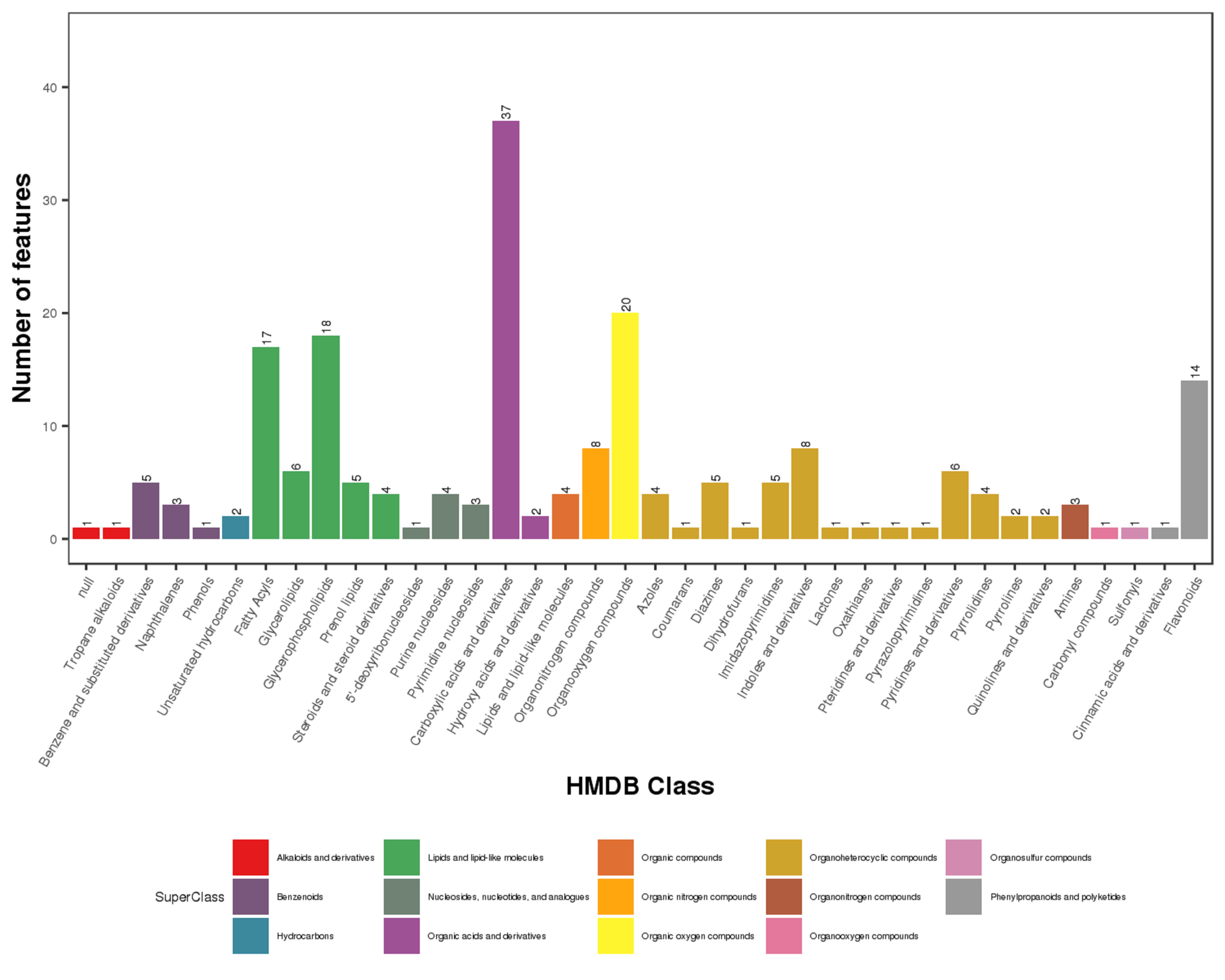

Supplement: Supplementary 1 — Fig. S1. The performance variation curves under different numbers of wavelengths selected by SPA in variety classification. Fig. S2. The performance variation curves under different numbers of wavelengths selected by SPA in GM status identification. Fig. S3. Metabolites detected and annotated in positive model according to Kyoto Encyclopedia of Genes and Genomes pathway classification. Fig. S4. Metabolites detected and annotated in negative model according to Kyoto Encyclopedia of Genes and Genomes pathway classification. Fig. S5. The NIR-related characteristics wavelength selection curves using guided backpropagation of CascadeSeed-2 for GM status identification. Fig. S6. The terahertz-related characteristics wavelength selection curves using guided backpropagation of CascadeSeed-2 for GM status identification. Data S1. Detail information of all metabolites detected in positive and negative model. [file plantphenomics.0071.f1.zip › Supplementary Figure S3.jpg]

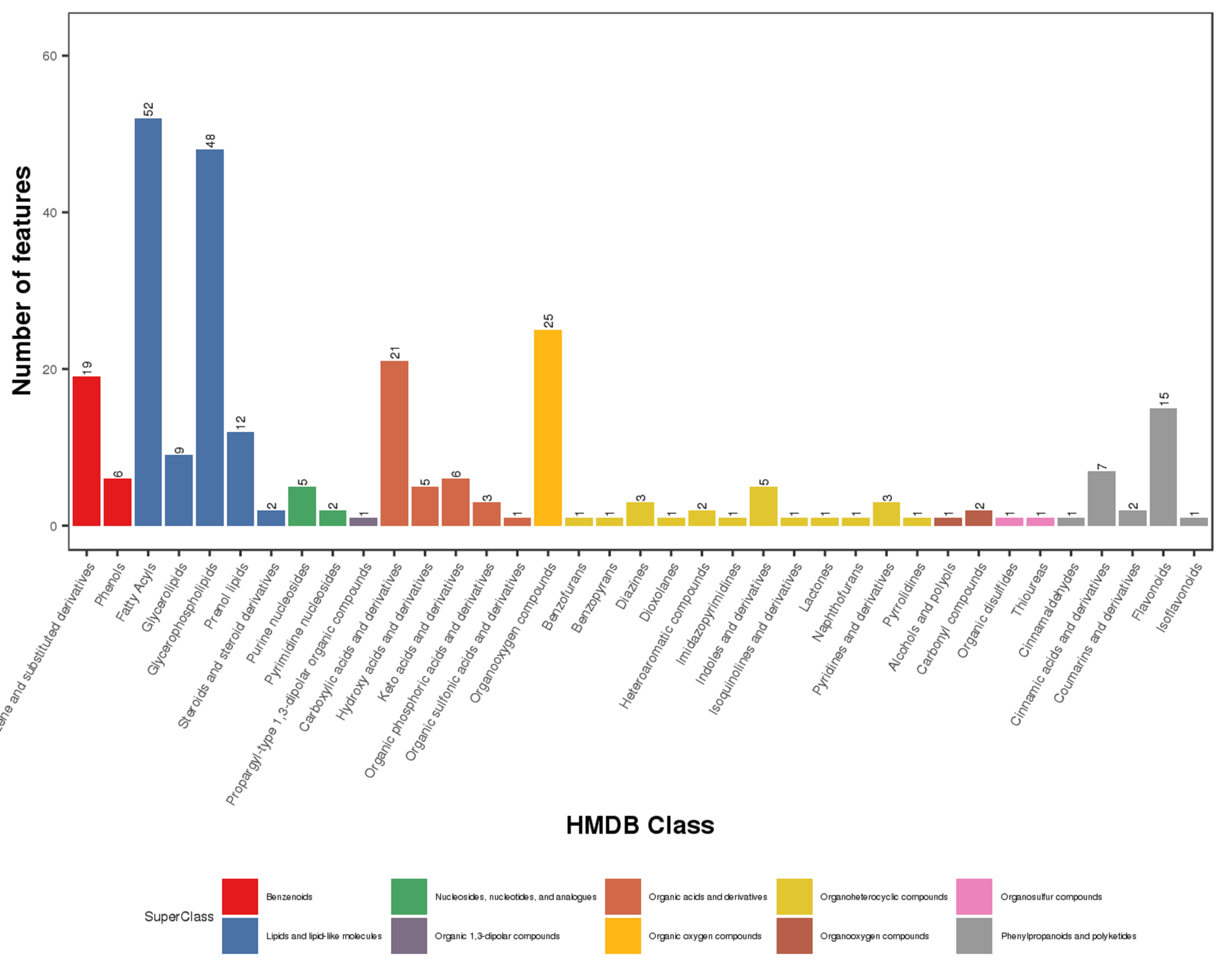

Supplement: Supplementary 1 — Fig. S1. The performance variation curves under different numbers of wavelengths selected by SPA in variety classification. Fig. S2. The performance variation curves under different numbers of wavelengths selected by SPA in GM status identification. Fig. S3. Metabolites detected and annotated in positive model according to Kyoto Encyclopedia of Genes and Genomes pathway classification. Fig. S4. Metabolites detected and annotated in negative model according to Kyoto Encyclopedia of Genes and Genomes pathway classification. Fig. S5. The NIR-related characteristics wavelength selection curves using guided backpropagation of CascadeSeed-2 for GM status identification. Fig. S6. The terahertz-related characteristics wavelength selection curves using guided backpropagation of CascadeSeed-2 for GM status identification. Data S1. Detail information of all metabolites detected in positive and negative model. [file plantphenomics.0071.f1.zip › Supplementary Figure S4.jpg]
